# A multiepitope peptide vaccine against HCV stimulates neutralizing humoral and persistent cellular responses in mice

**DOI:** 10.1186/s12879-019-4571-5

**Published:** 2019-11-05

**Authors:** Reham M. Dawood, Rehab I. Moustafa, Tawfeek H. Abdelhafez, Reem El-Shenawy, Yasmine El-Abd, Noha G. Bader El Din, Jean Dubuisson, Mostafa K. El Awady

**Affiliations:** 1 Micrbial Biotechnology Department, National Research Center, 33 Tahrir street, Dokki, Cairo, 12622 Egypt; 2 Univ. Lille, CNRS, Inserm, CHU Lille, Institut Pasteur de Lille, U1019 - UMR 8204 - CIIL- Centre d’Infection et d’Immunité de Lille, F-59000 Lille, France

**Keywords:** HCV, Vaccine, Humoral response, Cellular response

## Abstract

**Background:**

Although DAAs hold promise to significantly reduce rates of chronic HCV infections, its eradication still requires development of an effective vaccine. Prolonged T cell responses and cross neutralizing antibodies are ideal for vaccination against the infection. We aimed to design and synthesize a 6 multi epitope peptide vaccine candidate and provide evidence for production of extended cellular and neutralizing Abs in mice.

**Methods:**

Six peptides derived from conserved epitopes in E1, E2 (*n* = 2),NS4B, NS5A and NS5B were designed, synthesized in a multiple antigenic peptide (MAP) form and administered w/o adjuvant to BALB/c mice as HCVp6-MAP at doses ranging from 800 ng to 16 μg. Humoral responses to structural epitopes were assayed by ELISA at different times after injection. *ELISpot* assay was used to evaluate IFN ɣ producing CD4^+^/ CD8^+^ T- lymphocytes at extended durations i.e. > 20 weeks. Viral neutralization by mice sera was tested for genotypes 2a (JFH1) and a chimeric 2a/4a virus (ED43/JFH1) in HCVcc culture.

**Results:**

HCVp6-MAP confers potent viral neutralization and specific cellular responses at > 1600 ng/ animal for at least 20 weeks.

**Conclusion:**

We report on a promising anti HCV vaccine for future studies on permissive hosts and in clinical trials.

## Background

Hepatitis C Virus (HCV) infection is the main clinical entity leading to cirrhosis and hepatocellular carcinoma [1, 2]. Approximately 71 million patients are chronically infected worldwide [3]. Egypt has one of the global successful strategies of HCV control via enhanced screening, diagnosis and treatment protocols. Egypt had the highest global HCV epidemic witj 14% prevalence of chronic infection [4]. The global incidence of new infections is around 3–4 millions per year [2, 5], where not only developing but also developed countries contribute significantly as 18,000 new infections were recorded every year in the USA [6]. The use of direct-acting antivirals (DAAs) brought a new hope to beat such debilitating health problem, however, achieving HCV elimination is unlikely in absence of vaccines that diminish transmission of the infection. An ideal preventive vaccine should have the ability to induce cross neutralizing antibodies as well as extended and specific cytotoxic T lymphocytes (CTL) against HCV infected cells.

Envelope glycoproteins gpE1 and gpE2 of HCV are known to play active roles in cell entry. It was reported that the gpE1 region is more conserved and less immunogenic than gpE2, thus minimizing chances to generate neutralizing antibodies to gpE1 epitopes. Nevertheless, two monoclonal antibodies (mAbs) directed against the gpE1 peptide ranging from residues 313 to 327 have been identified as neutralizing antibodies [7]. On the other hand, gpE2 interacts with HCV receptor CD81 and is the major target of neutralizing antibodies. Indeed, gpE2 has a hypervariable region, called HVR1, at its N-terminus which contains a highly variable non-conformational neutralizing epitope [8, 9]. In addition, gpE2 envelope glycoprotein also contains two well-characterized non-conformational neutralizing epitopes, called epitopes I and II. Epitope I is located at residues 412–426 and is relatively conserved between different genotypes. Epitope II is located at positions 434–446 which varies among different genotypes. However, it seems that epitope II can also be a target of non-neutralizing antibodies which can potentially interfere with HCV neutralization [10]. We have previously shown that peptides derived from epitope II exerted potent interference with viral neutralization induced by epitope I [11]. It is noteworthy, that a majority of neutralizing antibodies targeting gpE2 recognize conformational epitopes located in the CD81-binding region. Moreover, AR3, a human neutralizing mAb, has been shown to contain discontinuous epitopes directed against three different regions in gpE2. These regions are 396–424, 436–447 and 523–540; the first and third regions also contribute to the CD81-binding domain of gpE2 [12].

On the other hand, it has been reported that T cell specific responses map to epitopes located within the nonstructural proteins of HCV [13]. Earlier data provided a strong evidence for the implication of the HCV specific CD4+ T lymphocyte response in HCV spontaneous clearance [14]. Furthermore, the depletion of CD4+ and CD8+ T lymphocytes was associated with persistent infection in chimpanzee [15, 16]. Moreover, the magnitude of IFN-γ secreted by HCV specific CD8+ T cells has been shown to be correlated with the HCV- RNA decline [17]. There is compelling evidence that the control of HCV infection requires a combination of a potent, specific and extended T cell response as well as broad neutralizing antibodies [18].

Several HCV vaccines have been designed, the majority are preclinical studies, while others include phases I or II clinical trials [19]. The vaccine candidates developed so far include: (i) recombinant proteins such as HCV core protein, non-structural proteins emulsified with MF59, HCV gpE1/E2 emulsified with MF59 [20], GI-5005: HCV NS3 and core proteins [21], HCV core protein/ISCOMATRIX [22]; (ii) synthetic peptides such as IC41 [23], a peptide (core) emulsified with ISA51 [24] and E1/E2 derived peptides [25]; (iii) DNA-based vaccine such as CIGB-230 [26] and others [27–29]; (iv) virus-based vaccine such as modified vaccinia Ankara virus-based HCV vaccine: TG4040 [30] recombinant adenoviral HCV vaccines [31], lentiviral vector-based HCV vaccine [32]. The majority of these strategies have limited efficacy for a number of reasons such as the delivery of a limited number of protective viral epitopes, the inclusion of incorrectly folded recombinant proteins, the limited humoral and cell-mediated responses that are associated with DNA vaccines, and the use of adjuvants with relatively poor potency [18, 33]. Moreover, there are several hurdles for the development of effective anti-HCV vaccine, namely hyper-variability of HCV genome, existence of multiple viral subtypes and quasispecies [34] and most importantly the absence of small animal model that provides statistically meaningful data upon challenging with the viral infection. The known adjuvants in several studies also displayed weak response in presenting the HCV epitopes.

In the current study, we designed 6 peptides derived from the most conserved regions of gpE1 (one peptide), gpE2 (2 peptides), NS4B (one peptide), NS5A (one peptide) and NS5B (one peptide). The amino acid sequences of the synthetic peptides were derived from HCV genotype 4 (ED43: GenBank accession no. Y11604) [35], the most prevalent genotype in Egypt (> 93%) [36, 37]. All peptides were synthesized using solid phase peptide synthesis (SPPS) technique in a multiple antigenic peptide form (8 arm MAP) to enhance immunogenicity of the peptides and avoid the use of the commercial adjuvants. The main aim of this study was to determine specific humoral and cellular responses against the selected 6 peptides prepared as a peptide mix and administered in 3 escalating doses in BALB/c mice. Most importantly the time course of both B and T cell responses were evaluated over more than 6 months post-vaccination.

## Methods

### Peptide design and synthesis

The three peptides derived from the envelope proteins gpE1 and gpE2 have been shown to possess B cell function as described previously in the epitope maps reported by Yusim, K. et al. (2005) [38] and by our laboratory [11, 25]. Whereas the 3 peptides derived from the nonstructural proteins (NS4B, NS5A, NS5B) have been shown to trigger HCV-specific CD4+ T cell response in subjects who spontaneously cleared the infection [13]. The peptides were synthesized by using the 9-fluorenylmethoxy carbonyl method. Each peptide was made up of an 8-branch multiple antigenic peptide (MAP) form where the arms are bound via a lysine backbone with 90% purity (Anaspec, Fremont, California, USA). The amino acid sequences in each peptide were aligned with viral isolates in HCV database to record the degree of sequence identity with HCV subtypes. The six peptide sequences used in the current study were derived from HCV genotype 4a (EG.ED43.Y11604). The alignment was done by using Clustal W multiple sequence alignment program at http://align.genome.jp/. Peptide sequences and alignments are summarized in Table 1.

**Table 1:** *Sequence identity of the selected peptide epitopes as compared with corresponding regions derived from genotypes 2a (JFH1) and a chimeric 2a/4a virus (ED43/JFH1)*

Protein	Peptide name	Amino acid position	Sequence alignment
E1	P315	a.a. 315–326	GHRMAWDMMMNW- Selected peptide Sequence GHRMAWDMMMNW- (ED43)/(JFH1) GHRMAWDMMMNW- JFH1
E2	P412	a.a. 412–423	QLINSNGSWHIN- Selected peptide Sequence QLINSNGSWHIN- (ED43)/2a (JFH1) QLIN**T**NGSWHIN- JFH1
E3	P517	a.a. 517–531	GTTDHVGVPTYDWGK- Selected peptide Sequence GTTDHVGVPTY**T**WG**E**- (ED43)/(JFH1) GTTD**RR**GVPTY**T**WG**E**- JFH1
NS4B	P1771	a.a. 1771–1790	GIQYLAGLSTLPGNPAIASL- Selected peptide Sequence GIQYLAGLSTLPGNPA**V**AS**M**- (ED43)/(JFH1) GIQYLAGLSTLPGNPA**V**AS**M**- JFH1
NS5A	P2121	a.a. 2121–2140	FFTEVDGIRLHRHAPKCKPL- Selected peptide Sequence FF**SW**VDG**VQI**HR**F**AP**TP**KP**F** - (ED43)/(JFH1) FF**SW**VDG**VQI**HR**F**AP**TP**KP**F**- JFH1
NSB5	P2941	a.a. 2941–2960	CGIYLFNWAVKTKLKLTP- Selected peptide Sequence CG**R**YLFNWAVKTKLKLTP- (ED43)/(JFH1) CG**R**YLFNWAVKTKLKLTP- JFH1

All lyophilized peptides were dsissolved in DMSO (Sigma, Germany) at a concentration of 10 μg/μL and stored at − 20 °C. Prior to immunization, peptides were diluted to the desired dose concentration and were kept at 4 °C.

### Selection of the epitopes

Selection of the Epitopes are based on three different criteria:

1. Their association with spontaneous clearance of viremia during acute infections.
2. Conservation of the sequence identity, especially among genotype 4 and other less frequent genotypes in Egypt .
3. Potential of each epitope to induce humoral and/or cellular immune responses as reported by other laboratories [13, 39, 40].
4. Peptides for CTL epitopes were selected on bioinformatics based calculations of the binding capacity to HLA molecules by using the program SYFPEITI (http://www.syfpeithi.de)

### Ethics statement

The mice (female BALB/c, 6–8 weeks old), weighing 18–20 g, were obtained from the animal house of the National Research Center in Egypt. Ethical approval was obtained from the Institutional Animal Care and Use Committee, NRC #17112). Animal care and handling were performed according to the guidelines set by the (WHO), Geneva, Switzerland and the MREC (Medical Research Ethics Committee), Cairo, Egypt [41]. Mice were maintained in a temperature controlled environment at 24 °C with a 12 h light/dark cycle, and were provided with drinking water and feed ad libitum. All efforts were taken to minimize the suffering of animals used. The mice were acclimatized in the laboratory conditions for a period of 1 week before being used in the experiment under observation to exclude any inter-current infection.

### Mice and immunization

Three groups were constituted, each consisting of 12 mice. Each mouse received a mixture of equal concentrations from each of the 6 peptides at a total dose of 800 ng (group 1), 1600 ng (group 2) and 16 μg (group 3).

Mice were immunized subcutaneously (S.C.) with a total volume of 120 μL containing either one of the above mentioned doses of the 6 peptide mixes in the MAP form (HCVp6) at weeks 0, 4, 8 and were bled for analysis through retro-orbital plexus/sinus before each injection and two weeks after each injection. Mice were kept for 20 weeks post-immunization and blood samples were withdrawn to test the persistence of cellular responses. The separated sera and PBMC’s were preserved at − 80 °C until tested. The control group (12 mice) received 120 μL PBS and were treated in the same way as groups 1–3. At 20 weeks post-immunization, mice were anaesthetized with diethyl ether and euthanized by cervical dislocation, then carcasses were incinerated. Immunization, sampling and testing protocol is shown in (Table 2).

**Table 2:** Immunization and sampling schedule of BALB/c mice

Weeks		0 W	2 W	4 W	6 W	8 W	10 W	20 W
Groups	Action	Pre-immunization analysis	Analysis	Boost1 & analysis	Analysis	Boost 2 & analysis	Analysis	Analysis
1 (*n* = 12) 800 ng HCVp6		800 ng ELISA & EliSpot	ELISA & EliSpot	800 ng ELISA, EliSpot & Neutralization	ELISA & EliSpot	800 ng ELISA & EliSpot	ELISA & EliSpot	EliSpot
2 (n = 12) 1600 ng HCVp6		1600 ng ELISA & EliSpot	ELISA & EliSpot	1600 ng ELISA, EliSpot & Neutralization	ELISA & EliSpot	1600 ng ELISA & EliSpot	ELISA & EliSpot	EliSpot
3 (n = 12) 16 μg HCVp6		16 μg ELISA & EliSpot	ELISA & EliSpot	16 μg ELISA, EliSpot & Neutralization	ELISA & EliSpot	16 μg ELISA & EliSpot	ELISA & EliSpot	EliSpot
control (n = 12) PBS 120 ul		PBS ELISA & EliSpot	ELISA & EliSpot	PBS ELISA, EliSpot & Neutralization	ELISA & EliSpot	PBS ELISA & EliSpot	ELISA & EliSpot	EliSpot

### Detection of humoral response by ELISA

Sera of the immunized mice receiving different doses of HCVp6 or PBS at different time points were tested for specific IgG AB against the followings; a) keyhole limpet hemocyanin (KLH) conjugated individual structural peptides representing the linear epitopes (KLH 315, KLH 412, KLH 517), b).mixture of KLH conjugated individual structural peptides and c) a mixture of 3 MAP peptides (MAP-mix) representing the conformational epitopes.

Briefly, ELISA plates were coated with 100 μL of individual structural peptides i.e. KLH 315, KLH 412, or KLH 517 (10 μg/mL). Additional plates were coated with 100 μL of 6MAP (10 μg/ml) which was prepared in 0.5 M carbonate/bicarbonate buffer (pH 9.6). The reason why humoral responses were not tested against non structural peptides is that the titers of antibody response to the 3 structural peptides used in the current immunogen was not different from the titer induced by 6 peptides including the above 3 structural plus 3 non structural peptides described herein. (results not shown).

Coated plates were incubated overnight at room temperature and washed with PBS containing 0.05% Tween 20 (PBS-T). Blocking buffer (2% Bovine serum albumin in PBS) was added and incubated for 2 h at 37 °C. After washing with PBS-T, diluted mice sera (1:1000 in PBS buffer containing 1% nonfat milk and 0.05% Tween-20) were added and incubated at 37 °C for two hours. Serial dilutions of mice sera, ranging from 1:50 to 1:2000 were tested in ELISA and showed that 1:1000 was the least serum concentration that elicited detectable Ab responses, results not shown). Plates were washed with PBS and incubated at 37 °C for two hours with Horseradish Peroxidase (HRP) conjugated Goat Anti-mouse IgG (1:2000; KPL, Maryland, USA). Detection was performed with O-Phenylene Diamine (OPD, 0.01%) substrate (Sigma, USA) for 30 min at 37 °C. Finally, the reaction was stopped using 3 M HCl and the absorbance was measured at 450 nm.

### Viral neutralization by the generated murine antibodies

The genotype 2a virus used in this study was based on the JFH1 isolate (genotype 2a; GenBank accession number AB237837) [42], kindly provided by T. Wakita (National Institute of Infectious Diseases, Tokyo, Japan). This virus contains mutations at the C terminus of the core protein leading to amino acid changes F172C and P173S, which have been shown to increase viral titers [43]. Furthermore, the N-terminal E1 sequence encoding residues *196* TSSSYMVTNDC has been modified to reconstitute the A4 epitope (SSGLYHVTNDC), as described previously [44]. In addition, neutralization experiments were also performed with a chimeric virus (ED43/JFH1), kindly provided by J. Bukh (Copenhagen University Hospital, Denmark), which is based on a JFH1 recombinant expressing core-NS2 of genotype 4a ED43 strain [45].

Neutralization experiments were performed as described previously [46]. Briefly, the 3 Antibodies (Abs) were diluted to final concentration of 1:50 and incubated with virus inoculums (containing 500 FFU/well) of JFH1 or ED43/JFH1 for 1 h at 37 °C. Ab/virus inoculum were added (50 μL/well) onto Huh-7 cells (1 × 10^4^ cells/well) [47], that were plated into a 96-well plate 24 h before. After 3 h of incubation at 37 °C and 5% CO2, the inoculum was replaced with 100 μL of complete medium followed by an additional incubation time of 27 h. Infected cells were fixed after 30 h with ice-cold methanol and stored at − 20 °C for 5 min at least. Immunofluorescence detection was performed with anti-E1 A4 mAb [48]for JFH1 infected cells and anti-NS5A Ab, which was kindly provided by M. Harris (University of Leeds, UK), for ED43/JFH1 infected cells. Both primary and secondary antibody incubations were carried out in PBS containing 10% goat serum or 10% horse serum respectively for 30 min at room temperature. For quantification of infection levels, images of randomly picked areas from each well were recorded and processed using Image J software. For each condition 12 pictures were counted, as we took 4 non-overlapping pictures per well working in triplicates. Cells labeled with anti-E1 mAb A4 or anti-NS5A were counted as infected cells. The total number of cells was obtained from DAPI-labeled nuclei. The infections were scored as the ratio of infected cells to total cells. Each experiment was performed 3 times in triplicates. Percentage neutralization was calculated in relation to the mean of non-immunized BALB/c mice serum/virus mix controls.

The 50% Inhibitory concentration (IC50) was determined against JFH-1 by performing serial dilutions of our antibodies in a similar manner to the inhibition assays. The percent neutralization was calculated as the percent reduction in Focus Forming Unit (FFU) normalized to virus incubated with non-immunized BALB/c mice sera. Data transformation and four-parameter nonlinear regression analysis were performed using GraphPad Prism software.

### Detection of IFN γ secreting CD4+ and CD8+ T lymphocytes

Peripheral blood mononuclear cells (PBMCs) were separated from mice blood using Ficol Hypaque and were then preserved in liquid N2 until assayed. A total of 20,000 mouse cells per well were collected equally from three animals (0.5 ml from each animal). Peptides were directly added to wells at a final concentration of 10 ng/mL.

The IFN γ secreting T lymphocytes were quantified using a mouse ELISPOT kit (Abcam, Cambridge, Massachusetts, USA). Briefly, 100 μL of PBS were added to 96-well plates pre-coated with an anti-IFN-γ mAb (MAHA S4510; Millipore). Twenty thousand cells per well were incubated in duplicate with 5 μg/mL of peptide or medium alone (Negative control wells) for 16 h in a 37 °C humidified incubator with 5% CO2. PBMCs from nonimmunized mice were stimulated with PHA and served as positive control. After removing cells and washing with wash buffer (PBS with 0.1% Tween 20, Sigma, Saint Louis, USA), 1:100 diluted biotinylated anti-IFN-γ were added and incubated for 90 min at 37 °C. After each incubation step, the plates were washed three times with (PBS-0.1% / Tween 20). After 1 h of incubation with streptavidin-alkaline phosphatase conjugate (1/5000 in PBS-0.1% Tween 20; Boehringer Germany), the plates were developed with a solution of 5-bromo-4-chloro-3-indolylphosphate (BCIP)-nitroblue tetrazolium (ready made provided with the kit) until blue spots appeared. Tap water was used to stop the reaction, and the plates were dried in air overnight. Individual spots were counted under a dissecting microscope.

### Statistical analysis

Comparisons were analyzed by one-way analysis of variance (ANOVA) with Post Hoc Tests (Duncan) and *P* value less than 0.05 was considered significant.

## Results

### Humoral immune responses to HCVp6

To evaluate the antibody titer against HCV p6, an in-house ELISA was used. Plates were coated with either individual envelope peptides linked to keyhole limpet hemocyanin (KLH) protein i.e.KLH-315, KLH-412 and KLH-517, a mixture of the 3 KLH-linked peptides (KLH-mix) or a mixture of 3 MAP peptides (MAP-mix). Sera of the three animal groups were tested for specific IgG titers.

Antibody titers towards the HCVp3 (structural peptides) and towards the HCVp6 (3 structural plus 3 nonstuctural peptides), revealed no significant difference in the ODs of HCVp3 compared to HCVp6). This might be attributed to the less immunogenicity of nonstructural peptides to induce B cells and also the low sensitivity of ELISA to detect such minor differences between HCVp3 and HCVp6.

**Fig. 1:**
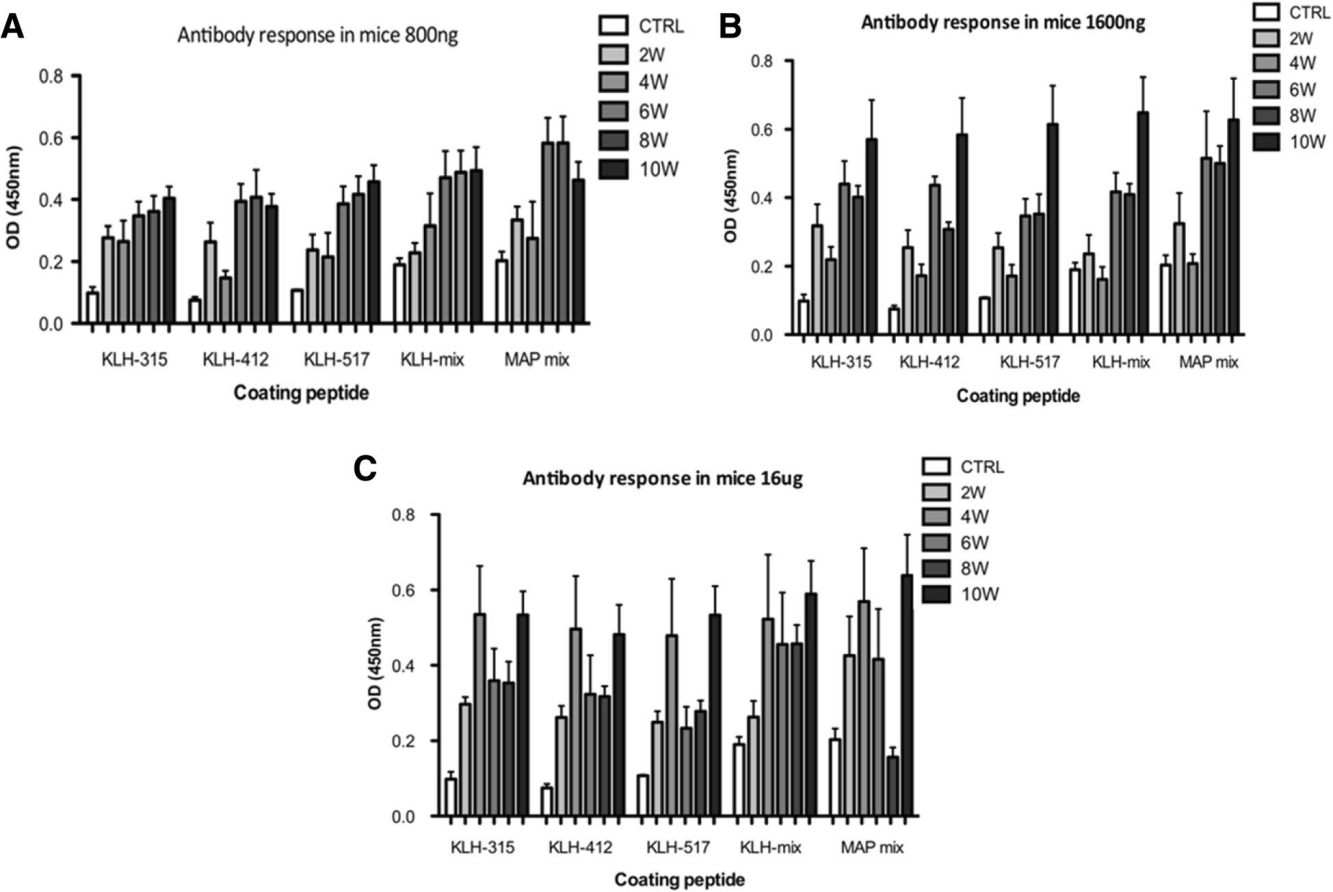
Humoral response to HCVp6 immunization Specific serum IgG response (expressed as OD) towards HCVP6 at different time points following mice immunization with 800 ng (**a**), 1600 ng (**b**) and 16 μg (**c**) of HCVP6. Peptides used for coating the plates are indicated in each figure. Serum samples of 3 animals from each time point were pooled and used for measuring IgG titers. Standard deviations are indicated above each column

The data depicted in Fig. 1 show that in groups A [800 ng] and B [1600 ng], the MAP mix displayed higher O. D values than individual peptides after 6–8 weeks when used for coating the plates. Finally, when using the dose 16 μg for immunization [group C], MAP mix displayed higher IgG antibody response than other immunizations only after 10 weeks. The drop in Ab response to 16 μg MAP mix after 8 weeks is not fully understood. As expected, when peptide mix (either KLH or MAP) was used for coating, the Ab reactivity displayed higher values compared with plates coated with individual peptides (*P* < 0.01). Based on this experiment, we selected MAP mix to study humoral response using a dose of 1600 ng HCVp6 throughout the period 6 to10 weeks post immunization.

In general, Ab reactivity tended to increase significantly with increasing the HCVp6 dose used for mice immunization i.e. Ab titers after 10 weeks of 16 μg HCVp6 were significantly higher (*P* < 0.01) than the group receiving 1600 ng which, in turn, was higher (P < 0.01) than the 800 ng group. Antibody production against the envelope peptides continued to be at the top levels (P < 0.01) until 10 weeks of immunization and remained at the same levels after 20 weeks of immunization (data not shown).

### HCV neutralization in vitro by murine antibodies

To test whether the antibodies generated in response to HCVp6 can neutralize HCV replication in Huh-7 cells [47], two viruses were used: the genotype 2a isolate JFH1 and a chimeric virus ((ED43/JFH1) based on a JFH1 recombinant expressing core-NS2 of genotype 4a ED43 strain in transfection of Huh-7.5 cells.

*Dose-dependent neutralization of JFH-1 HCVcc GT2a as determined by FFU reduction assay*

**Fig. 2:**
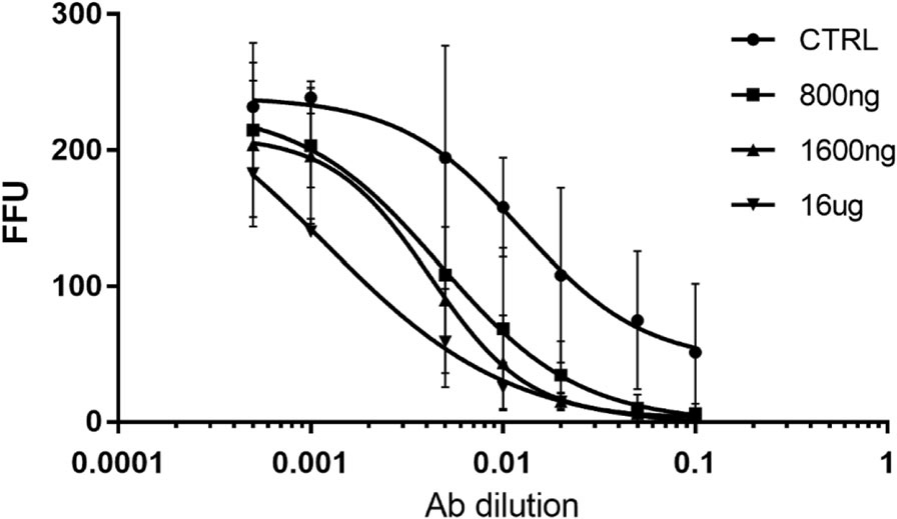
Dose-dependent neutralization of JFH-1 HCVcc GT2a as determined by FFU reduction assay. Infectious 2a JFH1 inoculum was incubated with each Ab, at dilutions ranging from 1:10 to 1:2000 against 2a JFH1, prior to inoculation onto Huh7 cells that were pre-seeded in 96 well plates. Cells were fixed and immunostained with A4 MAb at 30 h post infection, and counted by FFU-reduction assay. Each assay was performed in triplicates and data are shown as mean of three experiments. For each antibody, dose-dependent neutralization was measured to determine the concentration that resulted in a 50% reduction in FFU (IC50). For diluted IgG from mice immunized with 800 ng, 1600 ng and 16μg IC50 was calculated by nonlinear regression as a dilution of 004714, 0,004187 and 0,001185 respectively

The results depicted in Fig. 2 show that murine antibodies generated in response to HCVp6 at doses 800 ng, 1600 ng and 16 μg could completely neutralize the virus as determined by FFU reduction assay when used at a dilution of 10^− 1^. The neutralization capacity has shown gradual decrease with more dilution of mice sera in a clear dose dependent fashion. It is worth mentioning, that there is no difference between antisera derived from mice vaccinated with either dose of HCVp6. For each antibody, dose-dependent neutralization was measured to determine the concentration that resulted in a 50% reduction in FFU (IC50). For diluted IgG from mice immunized with 800 ng, 1600 ng and 16 μg, IC50 was calculated by nonlinear regression as a dilution of 0,004714, 0,004187 and 0,001185 respectively.

*Viral neutralization in Huh-7 cells infected with JFH1 isolate (genotype 2a)*

**Fig. 3:**
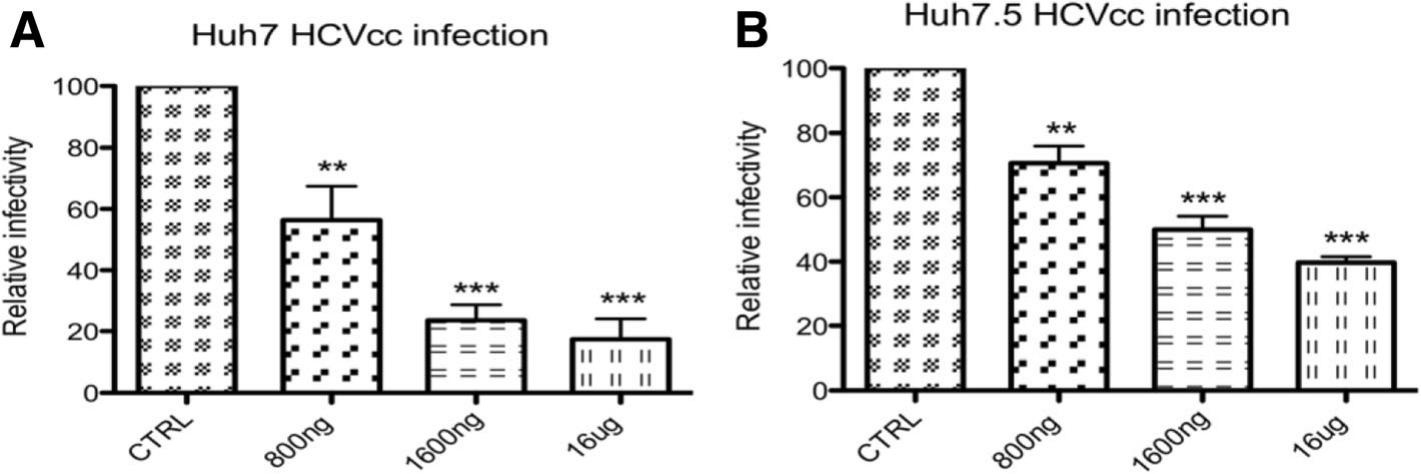
Neutralization of JFH1 genotype 2a (**a**) and chimeric virus (ED43/JFH1) based on a JFH1 recombinant expressing core-NS2 of genotype 4a ED43 strain (**b**) by murine antibodies generated in response to 3 doses of HCVp6. An Infectious virus inoculum pre-incubated with pools of immunized sera at a dilution of 1:50 were inoculated onto Huh-7 (**a**) or Huh-7.5 (**b**) cells. Infected cells were fixed at 30 h post-infection and processed for immunofluorescence to measure the number of residual infected cells. The data were normalized to parallel control experiments performed in the presence of non-immunized mice serum. Positive cells were scored as a ratio of infected cells to total cells as described in materials and methods. Diluted IgG from mice immunized with 800 ng,1600 ng and 16 μg HCVp6 as well as from controls were used for viral neutralization. The error bars are standard errors of the mean (SEMs) of results from 3 replicates

To test for the neutralizing activity of mice antibodies produced in response to HCVp6 immunization at 3 different doses, pools of sera were pre-incubated with JFH1 isolate (genotype 2a) followed by incubation with Huh-7 cells. Results of Fig. 3A show that murine anti HCVp6 Ab reduced the HCVcc infection as compared with controls in a dose dependent fashion with more neutralization of Abs raised against mice treated with 1600 ng [~ 70% inhibition] as compared to those treated with 800 ng [~ 40% inhibition]. Antibodies from mice treated with 16 μg HCVp6 reduced the HCV cc infection to less than 25% of controls [i.e. ~ 75% inhibition], It is noted that Abs raised by groups 2 and 3 had almost similar neutralizing capacities despite the large difference in the doses used for immunization (group 3 is 10-fold > group 2). Therefore 1600 ng is the optimum dose for subsequent studies.

*Viral neutralization in Huh-7.5 cells infected with ED43/ JFH1 chimera (genotype 4a/ 2a)*

To test for the neutralizing activity of mice antibodies produced in response to HCVp6 immunization at 3 different doses, pools of sera were pre-incubated with ED43/ JFH1 chimeric virus followed by incubation with Huh-7.5 cells. Results of Fig. 3B show that murine anti HCVp6 Ab reduced the HCVcc infection as compared with controls in a dose dependent manner with more neutralization of murine Abs that were generated in response to 1600 ng [~ 50% inhibition] than those responded to 800 ng [~ 25% inhibition]. Antibodies from mice treated with 16 μg HCVp6 reduced the HCVcc infection to around 40% of controls. Similar to neutralization assay of JFH1, 1600 ng HCVp6 is a suitable dose for subsequent experiments.

Of note, ED43 virus infectivity was neutralized to a lesser extent by our generated Abs than observed for the JFH1 strain, which will be discussed later.

### Cellular immune responses in immunized mice

**Fig. 4:**
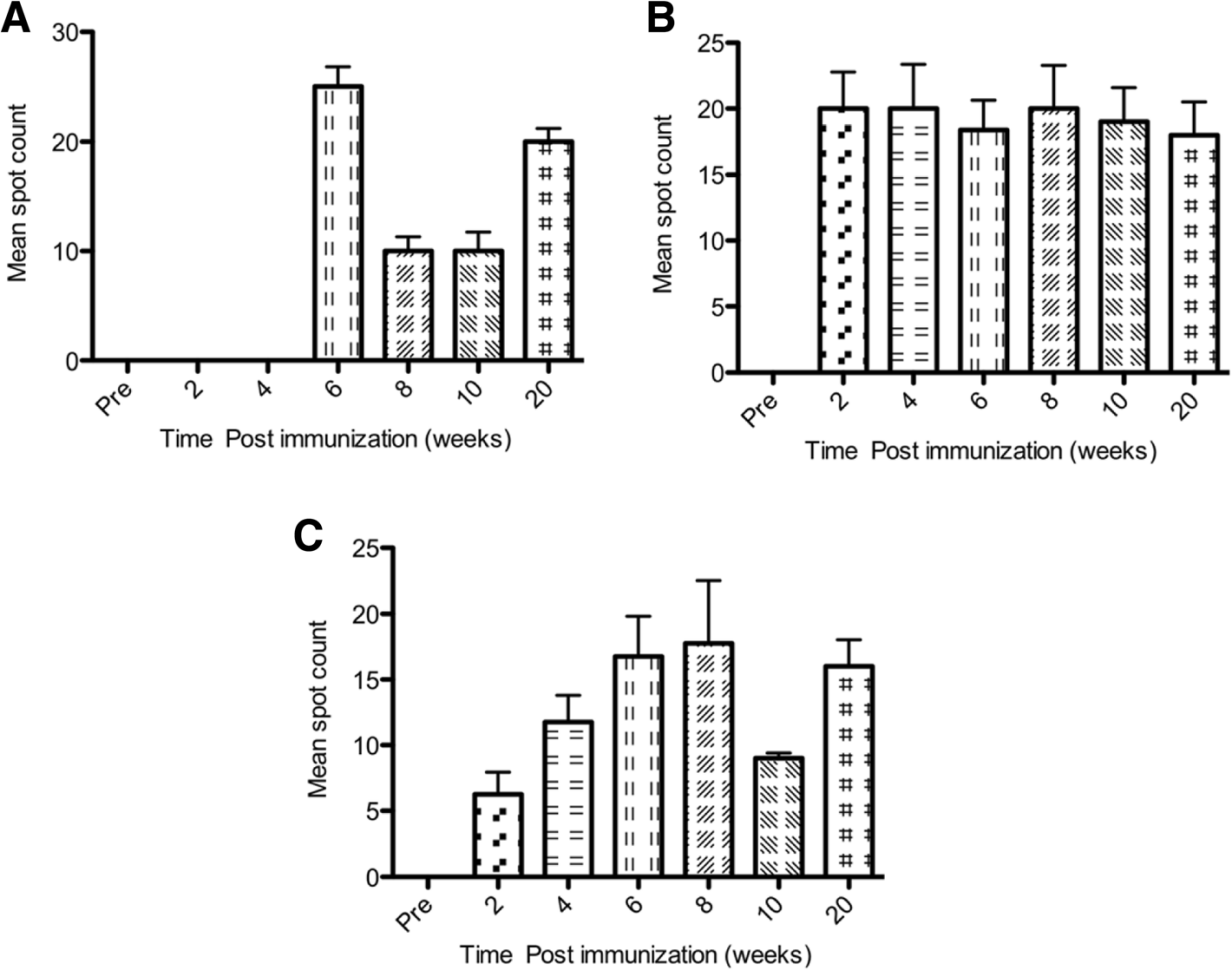
Murine cytokine production assay of PBMCs by Elispot in response to HCVp6. In vitro stimulation of cells derived from mice immunized with 800 ng (**a**), 1600 ng (**b**) and 16 μg (**c**) was performed with sc. HCVp6 immunization. Cells were pooled from 2 to 3 mice from each time point and allowed to react with anti IFN γ Ab followed by blue color development. Blue spots were counted and compared with controls. Standard deviations are shown above each column

Counts of IFN γ secreting CD4+ and CD8+ T lymphocytes in response to HCVp6 were determined using Elispot method in mice immunized with 800 ng, 1600 ng or 16 μg of HCVp6 at 2, 4, 6, 8 and 10 weeks post-immunization. To determine whether T cell response persists for longer periods, IFN γ secreting T lymphocytes were scored after 20 weeks post-immunization. The data displayed in Fig. 4 show that the T cell responses to the 3 doses of HCVp6 were significantly higher than the control group (i.e. non-immunized mice (*P* < 0.01). In group 1 (800 ng) the T cell response appeared after 6 weeks of immunization, then declined during the period from 6 to 10 weeks post-immunization then rose to moderate level after 20 weeks. In groups 2 and 3 (1600 ng and 16 μg) the cellular response began early after 2 weeks of immunization and stayed consistently throughout the entire period of the experiment, with less fluctuation of IFN γ secreting cell population in group 2 (1600 ng) than in group 3 (16 μg).

## Discussion

In absence of effective strategies to reduce annual rates of new HCV infections, research on the development of an HCV preventive vaccine remains a priority. An ideal preventive HCV vaccine should elicit neutralizing humoral as well as a persistent and specific CTL responses. The present findings revealed that murine immunity to HCVp6 vaccine candidate induced antibody as well as specific long lasting CTL responses. The strategy used in designing the current vaccine candidates is based on the selection of HCV epitopes that have been previously shown to induce potent CTL responses only in spontaneous clearers as well as to generate neutralizing antibodies that proved to play effective roles in viral clearance during acute HCV infection. Core specific epitopes were excluded since T-cell response magnitudes within core epitopes were similar in patients who spontaneously cleared HCV infection versus those who progressed to persistent infection [13]. In most cases, the selected epitopes were more or less genetically conserved across HCV genotypes. Wiesch et al. [13] reported that HCV-specific T cell epitopes are preferentially located in the nonstructural proteins. Furthermore, accumulated data support the importance of the cellular immune response in spontaneous clearance during acute HCV infection [49, 50]. Huang, et al. [40] demonstrated that the peptide vaccine vall 44 derived from two non-structural proteins and the core protein can enhance CD4+ and CD8+ cell subsets in HHD-2 mice and at in vitro level. The vall 44 displayed the ability to stimulate the IFN γ secreting cells in 30% of HCV chronically infected patients. During the acute phase of HCV infection, the cellular immune response plays a pivotal role in HCV clearance. Indeed, elevated counts of CD4+ and CD8+ T lymphocytes are closely associated with HCV elimination [51, 52].

On the other hand, peptides containing epitopes derived from the gpE1 and gpE2 glycoproteins have been shown to generate neutralizing antibodies [53]. Esumi et al. [54] reported that immunization with recombinant proteins derived from gpE1 and gpE2 together with HVR1 peptides derived from different isolates can confer a complete protection of chimpanzee challenged with the same isolate [54]. Peptides used in the current study have been recently shown to contain amino acid residues involved in CD81 blockade such as L413, G418, W420, G523, P525, Y527, W529 and G530 [55] Moreover, rabbits immunized with HVR1 derived peptides generated cross- reactive antibodies to HCV at high titers that blocked MOLT-4 infection with HCV [56]. In the present study, we have shown that the humoral arm of HCVP6 could efficiently neutralize HCV in vitro using two different genotypes, namely 2a (JFH1) and a chimeric virus 4a (ED43/JFH1). The reason why murine antibodies had more neutralization capacity for 2a as compared to 4a is not clearly understood. However, it is worth noting that HCV isolates have distinguishable neutralization-sensitive which are independent of genotype [57], and this might also explain that the genotype 4a strain used in our experiments was less sensitive to antibody neutralization than the genotype 2a strain.

The specificity of murine antibodies has been proven by a dose dependent assay which indicated that a vaccination with the least dose of HCVp6 i.e. 800 ng /mouse could achieve comparable neutralization efficiency to higher doses i.e. 1600 ng or 16 μg when anti sera were used at up to 1/2000 dilution in vitro*.*

A human mAb targeting gpE2 was shown to lower HCV titer in chronically infected chimpanzees [58]. Furthermore, HCV 1a derived gpE1/gpE2vaccine was protective against homologous or heterologous HCV 1a challenge in chimanzees [59]. This vaccine was approved for phase I clinical trial in humans. Recently, alteration in glycosylation pattern of gpE2 was shown to increase viral neutralization and confer protection against challenge in mice [60] However, HCV has the ability to mutate and produce new neutralizing antibody resistant viruses, a phenomenon that supports the notion that peptide vaccine which stimulates only the humoral response is not sufficient to achieve complete elimination of HCV. Furthermore, persistent infection leads to loss of CD4 helper T cell responses and compromises CD8 T cell activity with subsequent emergence of viral escape mutations in targeted CD8 T cell epitopes [61]. To overcome these mutational events we have focused, while selecting peptides, on amino acid stretches exhibiting relatively low variability among different HCV subtypes.

The current knowledge suggests that envelope glycoproteins are not totally devoid of T cell activity since at the level of clinical trials, the recombinant gpE1/gpE2 vaccine induced humoral and cellular responses in healthy volunteers [61–63].

Taken together the above findings, effective vaccine against HCV infection should elicit cellular immune response besides neutralizing antibodies. It was found that the NS5A protein inhibits the antiviral activity of the 2′-5′-OAS through direct interaction [64] and induces the IL8 production resulting in down regulation of the interferon stimulated gene expression [65], thus suggesting that NS5A plays a role in HCV immune evasion. Moreover, NS5A can bind to MYD88 and inhibits the TLRs signaling pathway [66] and hence blocks the IFN production [67]. We, therefore hypothesize that immunity against NS proteins can eliminate the cells that present non-structural epitopes in the context of major histocompatibility complex (MHC) class I, thus diminishes the ability of HCV to evade the immune system and potentiates viral clearance by the host. Although we have obviously neglected the fact that linear peptides may not retain their function within the naturally folded viral proteins, we thought, in the present study, that immune protection may be achieved via composing an immunogen made up of 6 MAP peptides; 3 genetically conserved/neutralizing antibody producing epitopes and 3 specific CD4+/CD8+ T lymphocyte stimulating epitopes. The findings presented in this study show that the 3 doses of non adjuvanted HCVp6 could elicit significant elevation of IgG titer throughout the 10 weeks of the experiment. Taken together, antibody production, viral neutralization and dose dependent studies indicate that the observed intracellular viral neutralization in vitro is a result of murine humoral immunity against HCVP- 6 while the inclusion of T cell epitopes in this vaccine can stimulate CTL activity. This activity displayed larger breadth of response and consistent stimulation of specific CD4+/CD8+ cells for more than 20 weeks post-immunization when using doses 1600 ng or 16 μg of the HCVp6 per mouse.

An increase in the IFN-γ gene expression was not detected by microarray or by quantitative PCR in the liver of chimpanzees chronically infected with HCV compared to uninfected chimpanzees [68], whereas IFN-γ mRNA expression levels in peripheral blood mononuclear cells of patients were significantly lower in non-responders to IFN α treatment compared to responders [69]. During the acute phase of HCV infection, the increased count of IFN-γ-producing HCV-specific CD8+ T cells is associated with virus elimination [70]. The isolated HCV-specific CD4+/ CD8+ T cells from chronically infected patients have been shown to display an impaired functional response including reduced cytotoxic potential, reduced secretion of Th1-type cytokines and a reduced proliferative capacity in response to ex vivo antigenic stimulation [71, 72]. In the liver of chimpanzees acutely infected with HCV, IFN-γ was detected only in animals displaying sustained or transient viral clearance in the context of an intra-hepatic HCV-specific T-cell response [73, 74]. Since IFN-γ is known to inhibit HCV genome replication in vitro [75], the current procedure based on EliSpot assay for scoring the IFN γ secreting cells provides reasonable experimental evidence on T cell efficiency.

## Conclusions

In summary, a peptide vaccine containing the B cell epitopes derived from the structural proteins and T cell epitopes from the non-structural region was designed and its immunogenicity was evaluated for its humoral and cellular efficiencies in a laboratory animal model. We conclude that the HCVp6 at a dose of 10 times dilution for any of the doses implemented is sufficient to block viral infection in HCVcc system. And the specific CD8+ cells produce high levels of IFN γ for extended periods and hence retain a potential function as CTL. After testing the safety profiles, this study opens the door for developing a well-tolerated and perhaps efficient vaccine that can progress towards clinical trials. A major limitation of this study is the lack of data on individual non structural peptides` stimulation of T- cells. A detailed analysis of this function is the subject of this laboratory’s next endeavor::

## Abbreviations

(CTL): cytotoxic T lymphocytes
(DAAs): direct-acting antivirals
(gp): Envelope glycoproteins
(HCV): Hepatitis C Virus
(HVR): hypervariable region
(KLH): keyhole limpet hemocyanin
(mAbs): monoclonal antibodies
(MAP): multiple antigenic peptide
(NR): non-structural protein
(PBMCs): Peripheral blood mononuclear cells
(S.C.): subcutaneously
